# Conventional Dendritic Cells and Slan^+^ Monocytes During HIV-2 Infection

**DOI:** 10.3389/fimmu.2020.01658

**Published:** 2020-08-13

**Authors:** Marco Iannetta, Stéphane Isnard, Jennifer Manuzak, Jean-Baptiste Guillerme, Mathilde Notin, Karine Bailly, Muriel Andrieu, Sonia Amraoui, Lene Vimeux, Suzanne Figueiredo, Bénédicte Charmeteau-de Muylder, Laura Vaton, Etienne X. Hatton, Assia Samri, Brigitte Autran, Rodolphe Thiébaut, Nathalie Chaghil, David Glohi, Charlotte Charpentier, Diane Descamps, Françoise Brun-Vézinet, Sophie Matheron, Remi Cheynier, Anne Hosmalin

**Affiliations:** ^1^Université de Paris, Institut Cochin, CNRS, INSERM, Paris, France; ^2^Sorbonne Université, Inserm 1135, Centre d'Immunologie et des Maladies Infectieuses, Cimi-Paris, Paris, France; ^3^INSERM, Univ. Bordeaux, CIC 1401, UMR 1219, Bordeaux Population Health Research Center, CHU Bordeaux, Bordeaux, France; ^4^Service des Maladies Infectieuses, AP-HP, Hôpital Bichat-Claude Bernard, Paris, France; ^5^INSERM, UMR 1137, IAME (Infection Antimicrobials Modelling Evolution), Université de Paris, Paris, France

**Keywords:** HIV-2, monocytes, slan^+^ monocytes, dendritic cells, cDC1, cDC2, controls

## Abstract

HIV-2 infection is characterized by low viremia and slow disease progression as compared to HIV-1 infection. Circulating CD14^++^CD16^+^ monocytes were found to accumulate and CD11c^+^ conventional dendritic cells (cDC) to be depleted in a Portuguese cohort of people living with HIV-2 (PLWHIV-2), compared to blood bank healthy donors (HD). We studied more precisely classical monocytes; CD16^+^ inflammatory (intermediate, non-classical and slan^+^ monocytes, known to accumulate during viremic HIV-1 infection); cDC1, important for cross-presentation, and cDC2, both depleted during HIV-1 infection. We analyzed by flow cytometry these PBMC subsets from Paris area residents: 29 asymptomatic, untreated PLWHIV-2 from the IMMUNOVIR-2 study, part of the ANRS-CO5 HIV-2 cohort: 19 long-term non-progressors (LTNP; infection ≥8 years, undetectable viral load, stable CD4 counts≥500/μL; 17 of West-African origin -WA), and 10 non-LTNP (P; progressive infection; 9 WA); and 30 age-and sex-matched controls: 16 blood bank HD with unknown geographical origin, and 10 HD of WA origin (GeoHD). We measured plasma bacterial translocation markers by ELISA. Non-classical monocyte counts were higher in GeoHD than in HD (54 vs. 32 cells/μL, *p* = 0.0002). Slan^+^ monocyte counts were twice as high in GeoHD than in HD (WA: 28 vs. 13 cells/μL, *p* = 0.0002). Thus cell counts were compared only between participants of WA origin. They were similar in LTNP, P and GeoHD, indicating that there were no HIV-2 related differences. cDC counts did not show major differences between the groups. Interestingly, inflammatory monocyte counts correlated with plasma sCD14 and LBP only in PLWHIV-2, especially LTNP, and not in GeoHD. In conclusion, in LTNP PLWHIV-2, inflammatory monocyte counts correlated with LBP or sCD14 plasma levels, indicating a potential innate immune response to subclinical bacterial translocation. As GeoHD had higher inflammatory monocyte counts than HD, our data also show that specific controls are important to refine innate immunity studies.

## Introduction

HIV-2 infection (1) is mostly prevalent in West Africa, and in populations emigrated from West Africa to Portugal and France (1–3). Eighty-seven per cent of the participants included in the French ANRS CO5 cohort originate from this geographical area. Compared to HIV-1 infection, HIV-2 infection is characterized by undetectable-to-low viremia, slower CD4^+^ T-cell decline and activation, slower disease progression (4, 5). The proportion of long-term non progressors (LTNP) is higher during HIV-2 than during HIV-1 infection (4). However people living with HIV-2 (PLWHIV-2) develop viral reservoirs with similar levels of HIV DNA in their PBMCs as people living with HIV-1 (PLWHIV-1) (6), and untreated HIV-2 infection can lead to AIDS, as current treatment options are scarcer than for HIV-1 infection (5).

Monocytes and conventional dendritic cells (DC) derive from common hematopoietic precursors. They are phagocytic, antigen-presenting cells and they secrete cytokines with effector, polarizing, inflammatory and reparatory functions. Monocytes represent 10–20% of peripheral blood mononuclear cells (PBMC). They are identified by their morphology and the expression of CD14. Among circulating monocytes, classical monocytes with a CD14^bright^, CD16^−^ phenotype represent the major population (90% of monocytes). Other monocytes, which will be called “inflammatory monocytes” along this text, express the FcγIII-receptor CD16, a receptor induced by microbial or inflammatory stimuli, conferring inflammatory, scavenger and cytotoxic functions. They comprise intermediate monocytes (CD14^bright^CD16^+^) and non-classical monocytes (CD14^+^, CD16^+^) (7). Circulating non-classical monocyte (CD14^+^CD16^+^) counts are higher in both asymptomatic chronically HIV-1-infected people with high viremia and in people developing AIDS than in people with low viremia or in uninfected donors (8, 9). They were found to be more permissive to HIV-1 infection *in vivo* and *in vitro* than other monocytes and to correlate with plasma sCD14 levels and microbial translocation (10, 11). Their accumulation is mostly due to a subpopulation of slan^+^ monocytes, inflammatory monocytes bearing the 6-Sulfo LacNAc (slan) glycosylation variant of the P selectin glycoprotein ligand 1 (PSGL-1) (12–14). Although these cells were originally described as a DC subpopulation, transcriptomic approaches have shown that they coclustered with monocytes and not with CD1c^+^ DCs (15, 16). Circulating slan^+^ monocytes produce higher TNF-α amounts than other monocyte subpopulations, and higher TNF-α amounts in HIV-1 viremic than in non-viremic people (13). Slan^+^ monocytes accumulate in viremic people (13) as well as in cART virosuppressed people who have experienced low CD4 count nadirs (14), but they are depleted during AIDS (17). They are known to be present in the lesions of major inflammatory chronic diseases like Crohn's disease, psoriasis or rheumatoid arthritis, which respond to therapeutic TNF-α blockade (18, 19). During HIV-2 infection, CD16^+^ monocyte numbers were shown to increase significantly among CD14^bright^ monocytes (20), but slan^+^ monocytes had never been specifically studied.

Myeloid or conventional DC (cDC), originally characterized as CD11c^++^CD14^−^ cells, negative for other lineage markers, are depleted from the circulation during HIV-1 infection (21). They were found to be progressively depleted from the circulation during chronic HIV-2 infection (20). They were further delineated into two major populations: cDC1 express the chemokine receptor XCR1, thrombospondin (CD141), and receptors for dead cells like Clec9, and are the equivalent of the murine CD8-α^+^ DC population, specialized in antigen cross-presentation to CD8^+^ T cells; cDC2 express the non-classical HLA Class I molecule CD1c as well as SIRP-α, and are believed to stimulate mostly CD4^+^ T lymphocytes (13, 22–25). Circulating cDC1 and cDC2 are both depleted during HIV-1 infection (13) and had not been specifically studied during HIV-2 infection.

During HIV-1 infection, the intestinal epithelium is durably damaged, allowing microbial product translocation into the circulation and the lymphoid system (26). This translocation has been associated with progression to non-AIDS-related complications like diabetes or cardiovascular diseases (26, 27). Plasma markers of bacterial translocation include lipopolysaccharides (LPS), major components of enteric Gram-negative bacterial outer membrane, which are classically measured in the plasma using the limulus assay but with low sensitivity and high variability. CD14, the main co-receptor for LPS detection, is shed from the surface of classical monocytes as a soluble molecule into the plasma. Microbial translocation and correlation with soluble CD14 (sCD14) levels were found in PLWHIV-1 from the USA, Canada or Finland (11, 26–28). In the SMART study on chronically HIV-1 infected participants, plasma sCD14 levels represented the only biomarker correlated with all-cause mortality, with a relative risk of 6 times in those with the highest quartile of CD14 levels, compared to those with the lowest quartile, after adjustment for CD4 cell counts, HIV-1 RNA levels and other inflammatory markers (29). CD163, a receptor involved in the clearance of hemoglobin, is also cleaved from monocytes or macrophages upon inflammation, leading to increased soluble CD163 (sCD163) plasma concentration. *In vitro*, it is expressed on M2-type monocytes stimulated with LPS and IL-4 or IL-13 (30). In PLWHIV-1, sCD163 plasma levels were found to be higher than in controls, to correlate positively with viral loads as well as with CD14^+^CD16^+^ monocyte counts, and inversely with the expression of CD163 on these cells and with CD4^+^ T cell counts. Also, sCD163 represents a marker of neurocognitive impairment or atheromatous plaque formation in people living with HIV (17, 31–33). LPS-binding protein (LBP), an acute-phase reactant secreted by epithelial intestinal or hepatic cells in response to inflammation, mediates binding of the Lipid A part of LPS to CD14. Circulating LBP levels correlate positively with plasma sCD14 levels during chronic HIV-1 infection (26, 27).

A recently uncovered particularity during HIV-2 infection is the lack of intestinal mucosa disruption, and the conserved balance between Th17 cells and regulatory T cells (34). Paradoxically, high levels of plasma sCD14 were found in the Portuguese HIV-2-infected cohort, as well as a significant activation of monocytes and DC, with high levels of HLA-DR and CD86 expression (20), like during HIV-1 infection (35), but LBP levels were similar to those of controls. In the ANRS CO5 HIV-2 cohort (*n* = 71), no elevation in plasma sCD14 was noted compared to healthy donors, but sCD14 levels correlated with IL-6 and high sensitivity C-reactive protein levels, and inversely with CD4^+^ T cell counts. Moreover, participants with plasma sCD14 levels >1.74 μg/mL had a 3.59 higher risk of disease progression than the others (*p* = 0.004), after adjustment for CD4 counts (36), like HIV-1 infected participants of the SMART study (29). sCD163 plasma levels were shown to decrease after cART in HIV-1-, and less in HIV-2-infected people from the Bissau HIV cohort (37, 38), but these data were not compared to those from healthy controls.

Here, we studied cDC1, cDC2 and monocyte subpopulations including slan^+^ monocytes, in HIV-2 infected participants from the IMMUNOVIR-2 study part of the ANRS-CO5 HIV-2 cohort, in correlation with viral loads and gut translocation markers. We distinguished long-term non-progressors (LTNP) and non-LTNPs (P, including progressive and intermediate evolutive profiles). Moreover, as PLWHIV-2 from the cohort were living in the Paris area, but originated mostly from West-Africa, we compared them not only to local Blood Bank healthy donors (HD), as usually done in many studies performed on HIV-2 infection, but also to local HD matched for their geographical origin, i.e., originating from West-Africa (GeoHD), in order to control the influence of ethnicity on our data.

## Results

### Participant Characteristics

All the participants were residents in the Paris area. Twenty-nine participants to the French ANRS-CO5 HIV-2 cohort, adult, asymptomatic and naïve of antiretroviral treatment were included into two groups depending on progression of HIV-2 infection: 19 Long-Term Non-Progressors (LTNP), including 17 from West-Africa, or 10 Non-LTNP (i.e., with progressive infection) (P), including 9 from West-Africa (Table 1). Long Term non progression was defined by asymptomatic infection for at least 8 years, with at least three CD4 cell counts or plasma viral load (pVL) measures during the 5 past years, stable CD4 cell counts (CD4) ≥ 500/mm^3^ since at least 5 years without a rapid decrease in the CD4 cell count slope (i.e., >50 cells/year) during the last 3 years). Thirty healthy donors, age- and sex- matched with the HIV-2^+^ participants, 16 from the French blood bank [Etablissement Français du Sang (EFS)] (HD), and 10 additionally matched with most HIV2^+^ participants for their West-African origin (GeoHD), were enrolled as control groups. Demographic and biological characteristics of HIV-2^+^ participants and HD are presented in Table 1. The HIV-2 P group was on average older (53 years) than the GeoHD group (42 years), a statistical difference not significant when considering only West African participants (Table 1). The sex-ratio varied from 0.78 to 1.83 between the groups. As expected, median CD4^+^ T cell counts were lower in HIV-2 P than in LTNP (in West African participants: 586 vs. 895 cells/μL *p* = 0.029).

**Table 1:** Demographic and clinical characteristics of the HIV-2-infected and uninfected donors participating in this study.

	**HD**	**GeoHD**	**HIV-2 LTNP**	**HIV-2 P**	***p***	**Dunn or Mann-Whitney *p***
*N*	16		19	10	NA	NA
*West Africans*	*NA*	*10*	*17*	*9*		
Age, years (median, [IQR])	50 [41–55]		50 [40–54]	53 [45–59]	**0.018 (K-W)**	GeoHD vs. P, *p* = **0.015**
		*42 [29–48]*	*50 [40–54]*	*53 [44–61]*	*0.069 (K-W)*	*GeoHD vs*. P, *p* = *0.06*
Sex (F/M), (ratio)	7/9 (0.78)		11/8 (1.38)	5/5 (1.00)	0.835 (Chi^2^)	
		*6/4 (1.50)*	*11/6 (1.83)*	*5/4 (1.25)*	*0.669 (Chi^2^)*	
Geographic origin	NA		2 France	1 France	0.316 (Chi^2^)	
		10 West Africa	17 West Africa	9 West Africa		
CD4, cell/μl (median [IQR])	NA	NA	895 [820–1203]	502 [423–858]		P vs. LTNP, *p* = **0.018**
			*895 [823–1187]*	*586 [356–936]*		P *vs. LTNP, p = **0.029***
Viral load, copies/ml (median [IQR])	NA	NA	<40 [<40–40], *n* = 17	<40 [<40–117], *n* = 9		

*HD, healthy donors from French blood bank (EFS); GeoHD, healthy donors of West-African origin; HIV-2 LTNP, long-term non-progressor HIV-2-infected donors; HIV-2 P, non-LTNP HIV-2 infected donors. Italics, West-African origin. NA, not available. IQR, interquartile range. K-W, Kruskall-Wallis. Chi^2^, Chi square test. Bold, p < 0.05*.

### Numbers and Percentages of Monocytes During HIV-2 Progressive vs. Non-progressive Infection Compared to Matched Controls From the Blood Bank or From West-African Origin

**Figure 1:**
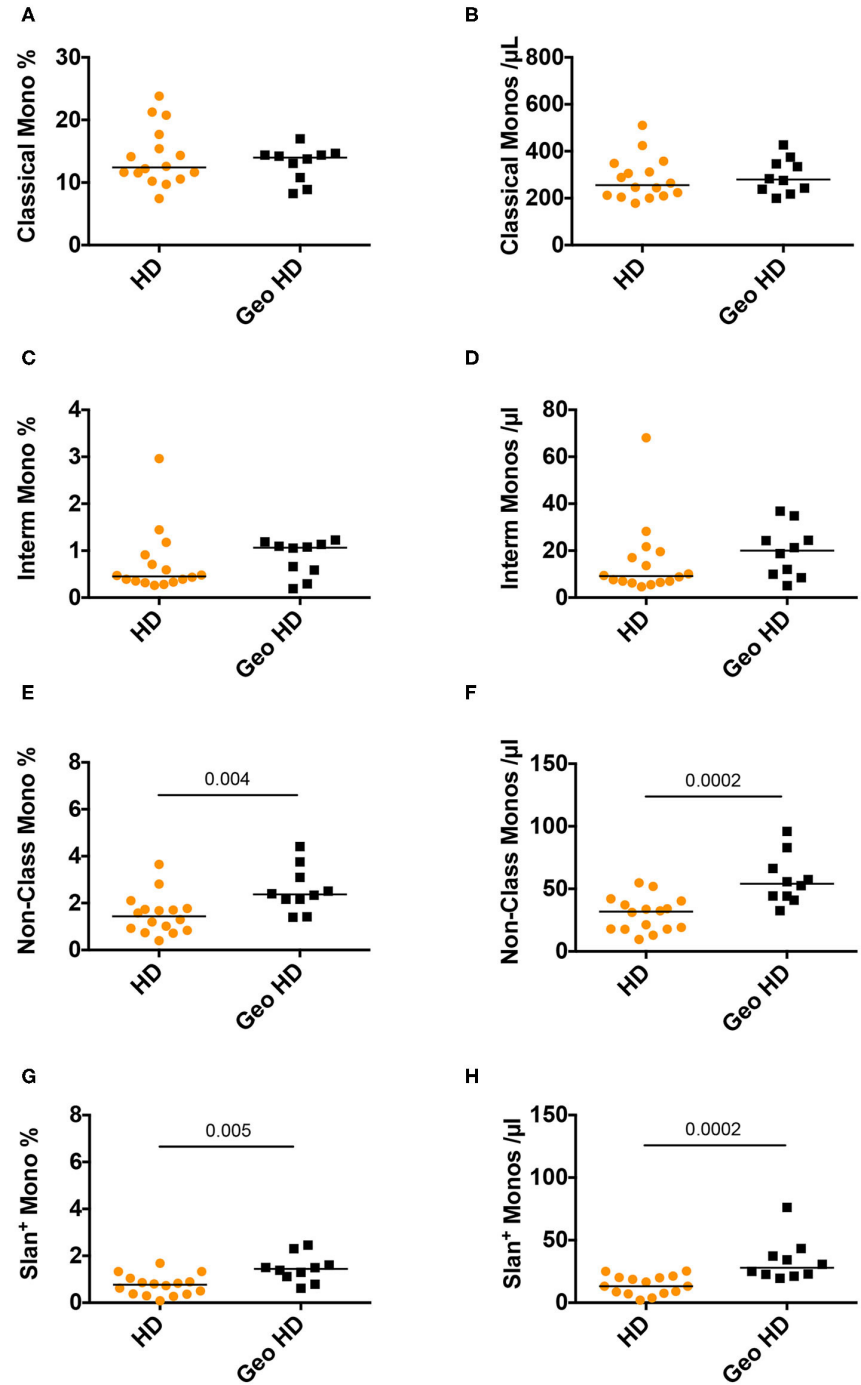
Monocyte subset percentages among CD45^+^ PBMC and absolute counts in controls from the blood bank (HD) compared to controls of West-African origin (GeoHD). **(A)** Classical monocyte (CD14^++^CD16^−^) percentages among CD45^+^ PBMC and **(B)** absolute counts/μL blood, **(C)** intermediate monocyte CD14^+^CD16^+^ percentages and **(D)** absolute counts / μL blood, **(E)** non-classical monocyte (CD14^±^*C*D16^+^) percentages and **(F)** absolute counts, **(G)** slan^+^ monocyte percentages and **(H)** absolute counts, in the two groups of age- and sex- matched healthy donors residing in the Paris area, one from the French Blood Bank (EFS) (HD) and the other of West-African origin (GeoHD). Orange: unknown origin, Black: West-African origin. Horizontal bars represent median values. Mann-Whitney's post-test *p* are represented above the horizontal line connecting the compared groups. Kruskall-Wallis tests and descriptive statistics can be found in Table 2 (in *italics* for West-African participants).

**Figure 2:**
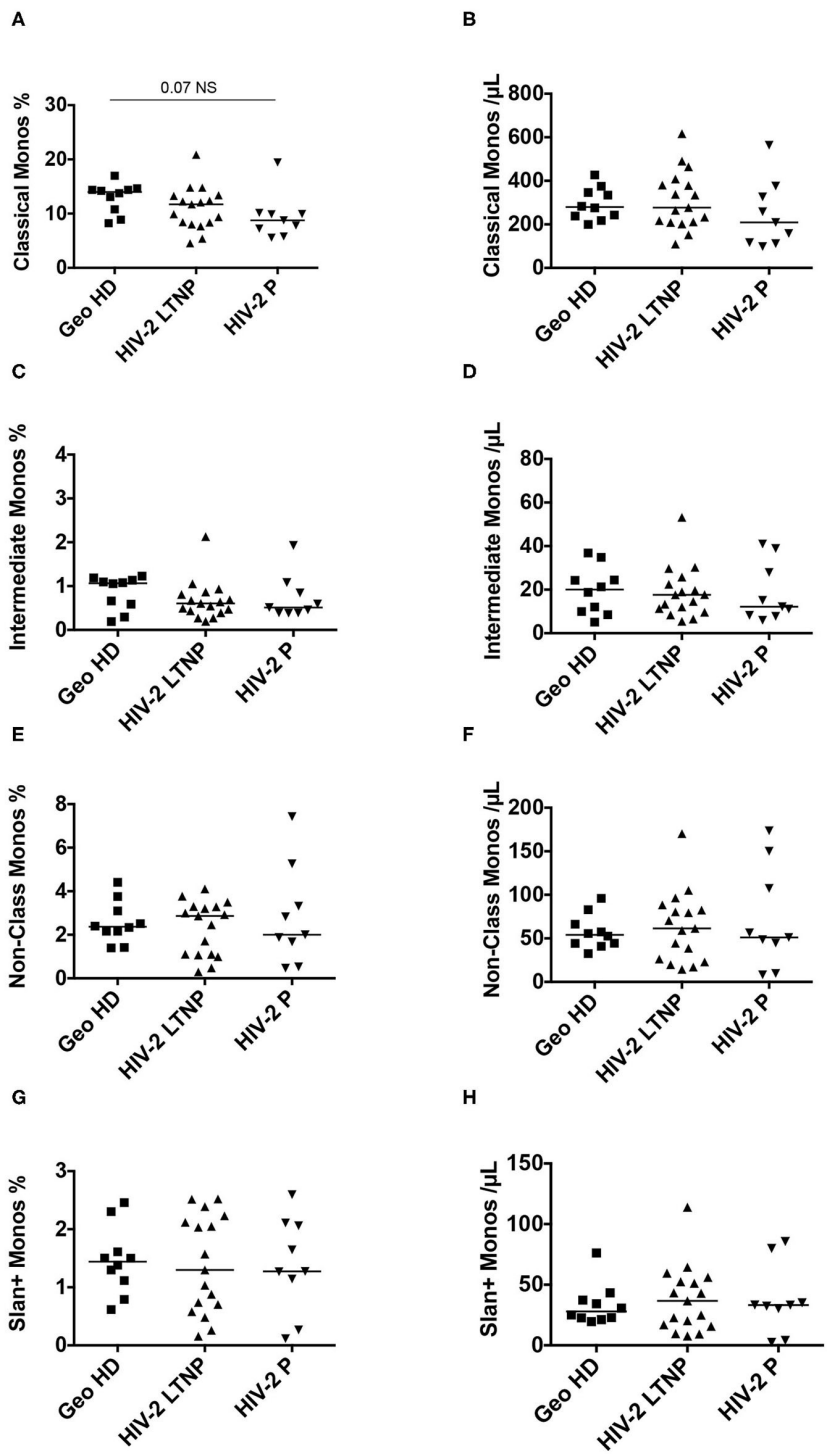
Monocyte subset percentages among CD45^+^ PBMC and absolute counts in HIV-2-infected participants (LTNP and P) and uninfected controls (GeoHD), all of West African origin. **(A)** Classical monocyte (CD14^++^CD16^−^) percentages among CD45^+^ PBMC and **(B)** absolute counts/μL blood, **(C)** intermediate monocyte CD14^+^CD16^+^ percentages and **(D)** absolute counts/μL blood, **(E)** non-classical monocyte (CD14^±^*C*D16^+^) percentages and **(F)** absolute counts, **(G)** slan^+^ monocyte percentages and **(H)** absolute counts, in GeoHD, HIV-2 LTNP and P groups, all of West-African origin. The data for the GeoHD group are the same as in Figure 1 Dunn's post-test *p* is represented above the horizontal line connecting the compared groups. Horizontal bars represent median values. Kruskall-Wallis tests and descriptive statistics can be found in Table 2, in *italics*.

Monocytes were classified according to their CD14 and CD16 expression (Supplementary Figure 1, CD14 vs. CD16 plot) into three subsets: classical monocytes (blue: CD14^++^CD16^−^), intermediate monocytes (gray: CD14^+^CD16^+^) and non-classical monocytes (yellow: CD14^+^CD16^±^), the latter expressing slan (yellow) or not (dark green). First, we compared monocyte subset relative and absolute counts in GeoHD (West-African origin) and HD (blood bank). The percentages and absolute counts of classical monocytes and intermediate monocytes were similar between GeoHD and HD (Table 2 and Figures 1A–D). Surprisingly, the percentages and absolute counts of non-classical monocytes were significantly higher in GeoHD than in HD (2.4 vs. 1.4%, Mann-Whitney test *p* = 0.004 and 54.1 vs. 31.8 cells/μL, *p* = 0.0002, respectively; Table 2 and Figures 1E,F). Slan^+^ monocyte percentages and absolute counts were significantly higher in GeoHD compared to HD (1.4 vs. 0.77%, *p* = 0.005 and 27.9/μL vs. 13.1/μL, *p* = 0.0002, respectively; Table 2 and Figures 1G,H). Thereafter, cell counts were compared only between participants of West-African origin: we compared GeoHD with HIV-2 LTNP (*n* = 17) and P (*n* = 9) of West-African origin (Table 2 and Figure 2). Classical and intermediate monocyte percentages and counts were similar between these groups (medians for classical monocytes: 14.0 in GeoHD, 12.1 in HIV-2 LTNP and 8.8 cells/μL in HIV-2 P), as well as the non-classical monocyte (54.1 in GeoHD, 61.5 in HIV-2 LTNP and 49.8 cells/μL in HIV-2 P) and the slan-monocyte counts (27.9 in GeoHD, 36.6 in HIV-2 LTNP and 32.8 cells/μL in HIV-2 P). This was surprising because when compared to HD from the local blood bank, as in (20), non-classical monocyte counts were twice as high in HIV-2 LTNP from all origins (*n* = 19; 61.5 vs. 31.8 cells/μL, Dunn's post test *p* = 0.01), and slan^+^ monocytes too (36.5 vs. 13.1, *p* = 0.004). In HIV-2 LTNP from West-African origin (*n* = 17), non-classical and slan^+^ cell counts were also significantly higher than in HD. No correlation of non-classical or slan^+^ monocyte counts was found with either VL or proviral loads (Supplementary Figure 2). Thus, non-classical monocytes and among them, slan^+^ monocyte counts were higher in GeoHD than in HD, but not between PLWHIV-2 and controls matched for geographical origin, indicating that the difference was related to ethnicity rather than to HIV-2 infection.

**Table 2:** Monocyte subset percentages among CD45^+^ PBMC and absolute counts in HIV-2- infected and uninfected donors.

		**HD**	**GeoHD**	**HIV-2 LTNP**	**HIV-2 P**	**Mann-Whitney *p* (HD/GeoHD)**	**Kruskal-Wallis *p* (GeoHD/LTNP/P)**
Classical Monocytes	%, Median [IQR]	12.4 [10.8–17.1]		12.1 [8.4–14.2]	9.3 [6.9–11.1]		**0.044**
			*14.0 [10.3–14.5]*	*12.06 [8.4–14.2]*	*8.8 [6.5–10.0]*	*0.957*	*0.066*
	Cell/μl, Median [IQR]	255 [210–339]		335 [211–464]	234 [115–339]		0.230
			*279 [233–354]*	*277 [210–394]*	209 [114–352]	*0.655*	*0.334*
Intermediate Monocytes	%, Median [IQR]	0.45 [0.34–0.86]		0.60 [0.44–0.86]	0.49 [0.39–0.90]		0.540
			*1.07 [0.52–1.15]*	*0.60 [0.42–0.84]*	*0.51 [0.39–0.96*]	*0.240*	*0.299*
	Cell/μl, Median [IQR]	9.14 [6.66–18.91]		17.7 [11.3–25.7]	11.6 [7.7–30.6]		0.629
			*20.0 [9.62–27.1]*	*17.6 [10.5–24.1]*	*12.2 [8.0–33.4]*	*0.121*	*0.905*
Non-classical Monocytes	%, Median [IQR]	1.4[0.9–1.8]		2.86 [1.1–3.3]	2.0 [1.4–3.8]		0.955
			*2.4 [2.0–3.3]*	*2.86 [1.1–3.3]*	*2.0 [1.1–4.3]*	***0.004***	*0.937*
	Cell/μl, Median [IQR]	31.8 [17.9–39.6]	*54.1 [43.4–70.5]*	61.5 [26.3–82.4]*61.5 [24.6–85.4]*	49.8 [30.0–118] *49.8 [18.5–94.6]*	***0.0002***	0.820 *0.961*
Slan+ Monocytes	%, Median [IQR]	0.77 [0.37–1.00]		1.3 [0.7–2.1]	1.3 [0.9–2.1]		0.981
			*1.4 [1.0–1.8]*	*1.3 [0.6–2.2]*	*1.3 [0.7–2.1]*	***0.005***	*0.943*
	Cell/μl, Median [IQR]	13.1 [7.6–20.1]		36.6 [16.9–52.4]	32.8 [18.6–46.3]		0.813
			*27.9 [22.3–38.8]*	*36.6 [16.3–54.3]*	*32.8 [10.7–34.6]*	***0.0002***	*0.967*

*HD, healthy donors from French blood bank (EFS); GeoHD, healthy donors of West-African origin; HIV-2 LTNP, long-term non-progressor HIV-2-infected donors; HIV-2 P, non-LTNP HIV-2 infected donors. Italics, West-African origin, as in Figure 2. IQR, interquartile range. Bold, p < 0.05*.

### Numbers and Percentages of Conventional DC During HIV-2 Progressive vs. Non-progressive Infection Compared to Matched Controls From the Blood Bank or of West-African Origin

Conventional DC were characterized according to the preferential expression of the surface markers CD141 (BDCA3) on cDC1 or CD1c (BDCA1) on cDC2, among CD14low, CD16^−^, HLA-DR^+^, CD19^−^ cells (Supplementary Figure 1). Percentages among total CD45^+^ PBMC and absolute counts per μL blood for the two subsets of cDC were compared among the different groups of HIV-2-infected and uninfected individuals.

#### cDC1

**Figure 3:**
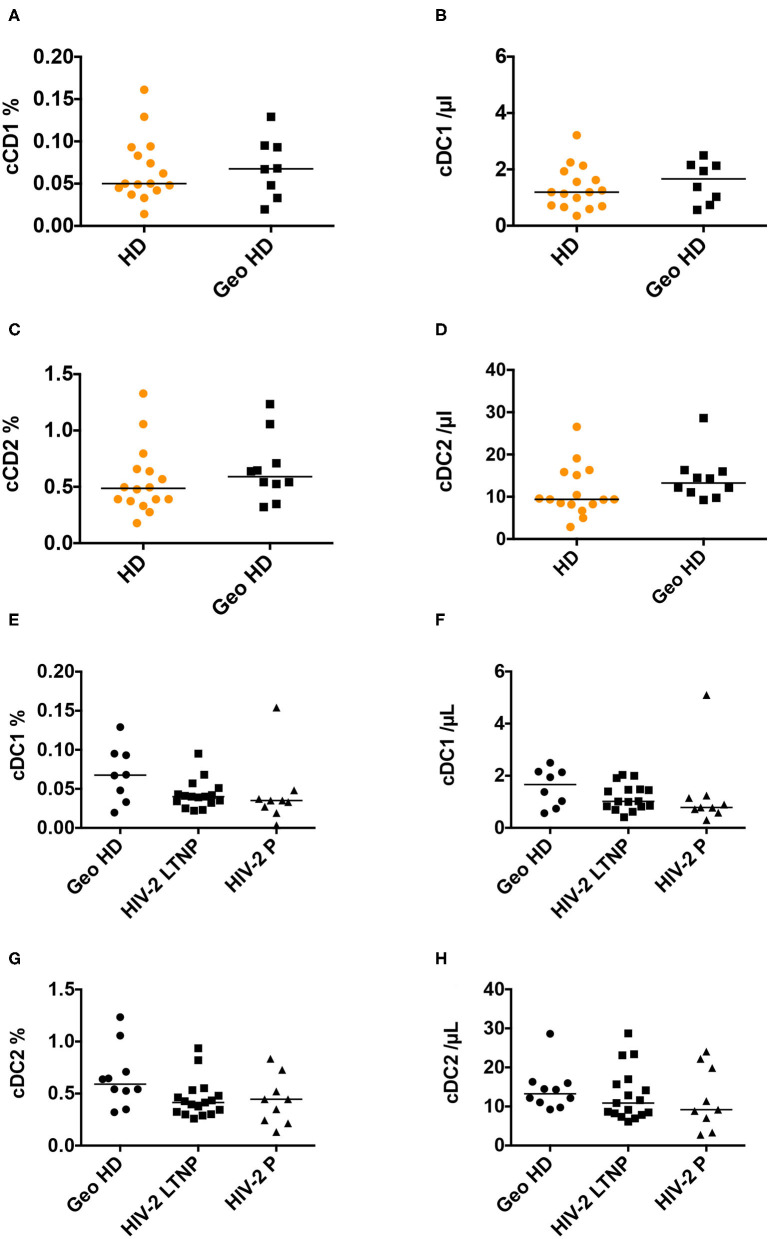
Conventional cDC1 and cDC2 percentages among CD45^+^ PBMC and absolute counts. **(A)** Conventional DC1 percentages among CD45^+^ PBMC and **(B)** absolute counts/μL blood, **(C)** cDC2 percentages and **(D)** absolute counts/μL blood, in HD and GeoHD. Orange: unknown origin, Black: West-African origin. **(E)** cDC1 percentages and **(F)** absolute counts, **(G)** cDC2 percentages and **(H)** absolute counts, in GeoHD, HIV-2 LTNP and P, all of West-African origin. Horizontal bars represent median values. Mann-Whitney tests, Kruskall-Wallis tests and descriptive statistics can be found in Table 3 (in *italics* for West-African participants).

Conventional DC1 percentages were similar between HD and GeoHD (Table 2, Figures 3A,B) as well as between PLWHIV-2 and GeoHD (Table 2, Figures 3E,F). Without one outlier participant, cDC1 percentages would have been lower in HIV-2 P than in GeoHD (0.034 vs. 0.068% Dunn's post-test *p* = 0.029, Figure 3E), cDC1 absolute counts followed a similar tendency (0.78 vs. 1.66 cells/μL, Dunn's *p* = 0.066, Figure 3F).

#### cDC2

Conventional cDC2 percentages and counts were similar between HD and GeoHD and between PLWHIV-2 and GeoHD (Table 3, Figures 3C,D,G,H).

**Table 3:** Conventional cDC1 and cDC2 percentages among CD45^+^ PBMC and absolute counts in HIV-2-infected and uninfected donors.

		**HD**	**GeoHD**	**HIV-2 LTNP**	**HIV-2 P**	**Mann-Whitney *p* (HD/GeoHD)**	**Kruskal-Wallis *p* (GeoHD/LTNP/P)**
cDC1	Median [IQR], %	0.050 [0.043–0.091]		0.040 [0.032–0.045]	0.034 [0.025–0.040]		0.060
			*0.068 [0.043–0.091*]	*0.040 [0.033–0.049]*	*0.035 [0.023–0.042]*	*0.776*	*0.119*
	Median [IQR], cell/μl	1.190 [0.700–1.850]		1.020 [0.786–1.470]	0.777 [0.564–1.170]		0.086
			*1.66 [0.81–2.15]*	*1.020 [0.820–1.470]*	*0.782 [0.650–1.19]*	*0.444*	*0.213*
cDC2	Median [IQR], %	0.487 [0.377–0.653]		0.414 [0.302–0.533]	0.409 [0.236–0.573]		0.425
			*0.590 [0.482–0.797]*	*0.414 [0.314–0.507]*	*0.446 [0.229–0.625]*	*0.280*	*0.066*
	Median [IQR], cell/μl	9.38 [8.21–15.68]		10.90 [7.85–16.98]	9.03 [6.11–20.44]		0.702
			*13.25 [10.74–16.06]*	*10.90 [8.05–16.33]*	*9.19 [5.18–21.02]*	*0.084*	*0.336*

*HD, healthy donors from French blood bank (EFS); GeoHD, healthy donors of West-African origin; HIV-2 LTNP, long-term non-progressor HIV-2-infected donors; HIV-2 P, non-LTNP HIV-2. Italics, West-African origin, as in Figures 3E–H. IQR, interquartile range*.

### Effect of Sex on the Numeration of Monocytes and DC

As men and women have different innate immune responses (39), and as there were differences in gender ratios between the different groups of donors, we compared between female and male individuals the numerations of these populations where significant differences in myeloid cell counts were noted. Supplementary Figure 3 shows that there was no difference according to participant's sex for classical monocyte percentages or slan^+^ monocyte counts, but that the median count of non-classical monocytes was higher in women than in men (53 vs. 38 cells/μL, Mann Whitney's *p* = 0.02), which might bear on the medians obtained for West-African LTNP whose female/male ratio was the highest.

### Correlation Between Bacterial Translocation and Myeloid Activation

**Figure 4:**
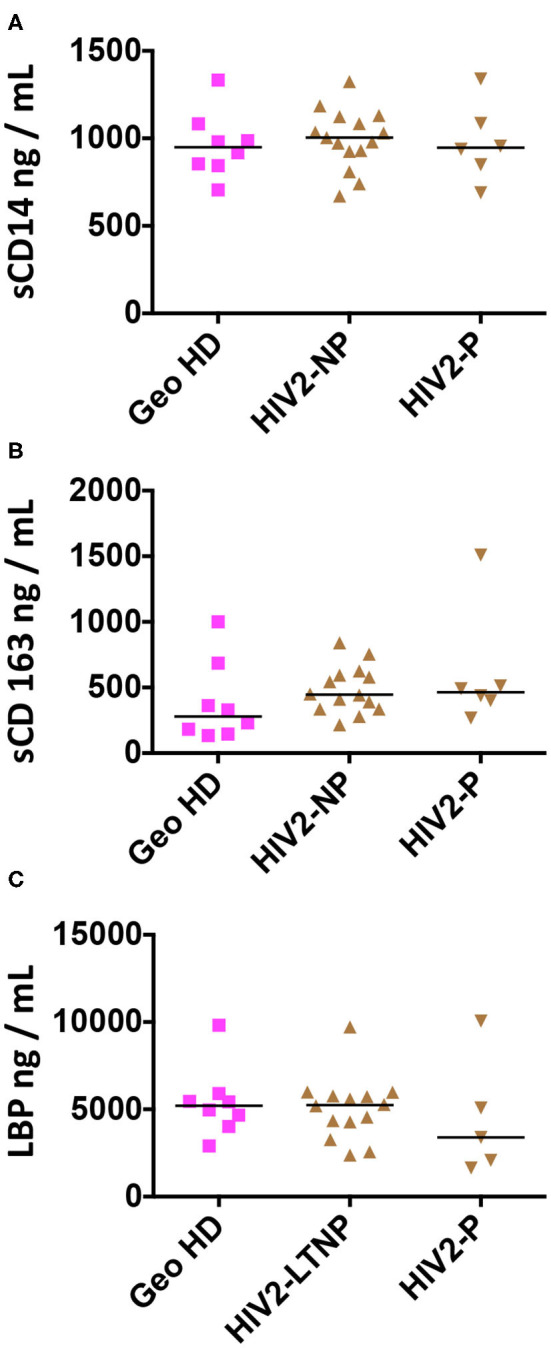
Plasma concentrations of sCD14, sCD163 and LBP in HIV-2-infected participants and uninfected controls (GeoHD), all of West African origin. Plasma concentrations of **(A)** sCD14, **(B)** sCD163, and **(C)** LBP. Horizontal bars represent median values. Kruskall-Wallis test and descriptive statistics can be found in Table 4.

Plasma markers of bacterial translocation and myeloid activation sCD14, sCD163, and LBP were measured in the plasma from HIV-2-infected people and GeoHD. As shown in Table 4 and Figure 4, there was no difference in the levels of these soluble markers between the groups. In addition, we measured the plasma concentrations of GM-CSF, an inflammatory cytokine produced by myeloid and lymphoid cells. GM-CSF plasma levels can be higher, but moderately, in asymptomatic chronically HIV-1-infected people than in controls (40), and can be high during acute HIV-1 infection, predicting a lower viral setpoint (41). *In vitro*, GM-CSF induces the differentiation of classical monocytes into slan^+^ monocytes (13). In the plasma from all of the HIV-2 infected donors and African individuals studied here, GM-CSF levels were normal, i.e., below 4 pg/mL, the detection limit of the test (except GeoHD #17 at 6 pg/mL, who had the second highest slan^+^ -monocyte count in this group at 43 cells/μL).

**Table 4:** Comparison of sCD14, sCD163 and LBP plasma levels in HIV-2 infected participants of West-African origin and GeoHD.

**Group**		**GeoHD**	**HIV-2 LTNP**	**HIV-2 P**	**Kruskal-Wallis *p***
sCD14	ng/mL, Median [IQR]	*949* *[847–1,059]*	*1,004* *[926–1,124]*	*938* * [769–1,020]*	*0.504*
sCD163	ng/mL, Median [IQR]	*280* * [155–605]*	*446* * [336–603]*	*490* * [352–1,010]*	*0.210*
LBP	ng/mL, Median [IQR]	*5,200* * [4,185–5,793]*	*5,241* * [4,031–5,822]*	*2,734* * [1,755–8,385]*	*0.437*

*GeoHD, healthy donors; HIV-2 infected donors; HIV-2 LTNP, long-term non-progressor HIV-2-infected donors; HIV-2 P, non-LTNP HIV-2 infected donors. All donors of West-African origin, as in Figure 4. Italics, West-African origin. IQR, interquartile range*.

**Figure 5:**
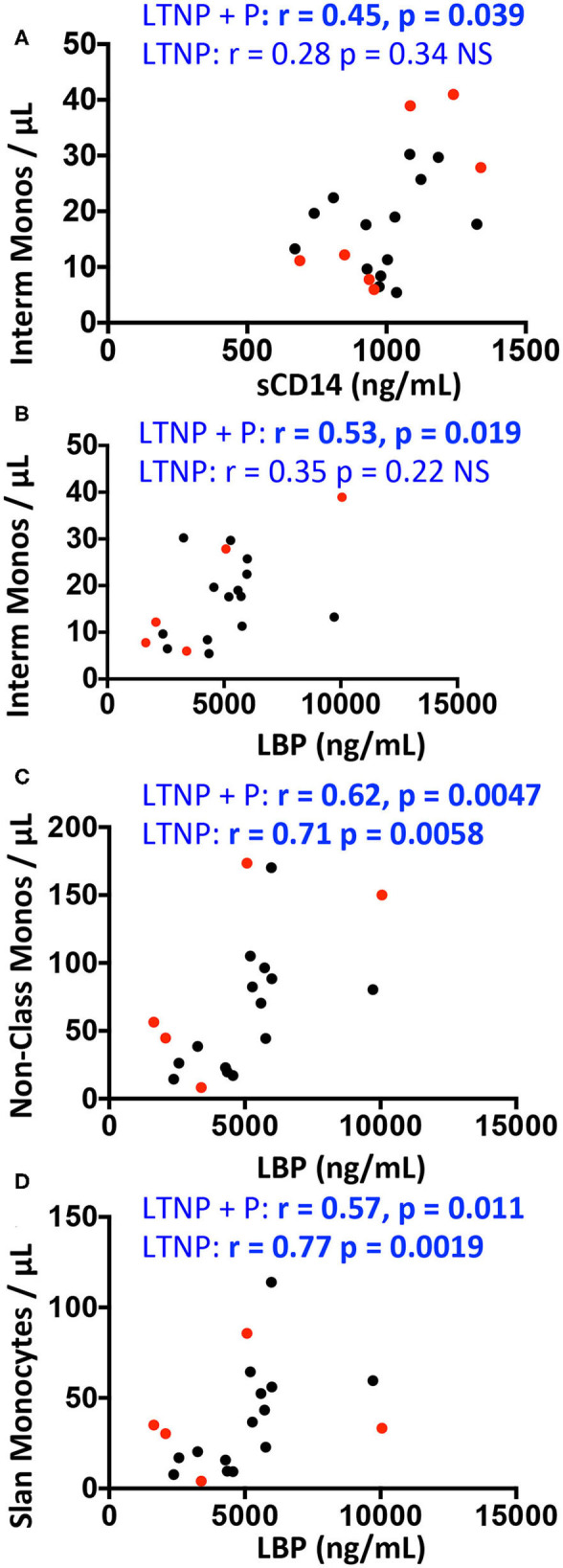
Correlations between monocyte counts and plasma concentrations of sCD14, sCD163, and LBP in HIV-2 infected participants and uninfected controls (GeoHD), all of West-African origin. Data from PLWHIV-2 from Figure 2 were correlated to data from Figure 4 by Spearman's test. The significant correlations are depicted. **(A)** Intermediate monocytes/μL vs. sCD14 plasma concentration. **(B–D)** Intermediate, non-classical or slan monocytes/μL vs. LBP. Correlations are given for all PLWHIV-2 (LTNP + P) or for LTNP alone. Progressor PLWHIV-2 (P) are indicated in red.

Correlations between plasmatic markers of bacterial translocation and myeloid cell counts within each group are shown in Figure 5 for West African participant samples. A positive correlation was observed between intermediate monocyte counts and both sCD14 and LBP plasma levels in all HIV-2-infected donors (LTNP+P) (Spearman correlation *r* = 0.45, *p* = 0.039, and *r* = 0.53, *p* = 0.019, respectively, Figures 5A,B) but not in LTNP donors alone. Similarly, LBP levels correlated positively with non-classical monocytes (*r* = 0.62, *p* = 0.0047) and with slan^+^ monocytes (*r* = 0.57, *p* = 0.011; Figures 5C,D) in LTNP + P; this correlation was sustained in LTNP alone (*r* = 0.71, *p* = 0.0058 and *r* = 0.77, *p* = 0.0019; Figures 5C,D).

## Discussion

Our work establishes, in untreated HIV-2 infected participants from the IMMUNOVIR-2 study, the blood counts of sub-populations of monocytes or cDC that were shown previously to be affected in HIV-1 infected individuals. PLWHIV-2 from this study lived in the Paris area, and originated mostly from West-Africa like the majority of PLWHIV-2. Therefore, to control for the influence of ethnicity on our data, we compared the counts from PLWHIV-2 of West-African origin not only with those from age- and sex-matched donors from the Blood Bank (HD), but also to those from age- and sex-matched healthy donors of West-African origin (GeoHD). The percentages and counts of circulating inflammatory non-classical monocytes, and especially slan^+^ monocytes, were higher in GeoHD than in HD. Thus, even if the counts of circulating inflammatory non-classical monocytes, and especially slan^+^ monocytes, were higher in long-term non progressor (LTNP) PLWHIV-2 than in HD, they were not significantly different from those of GeoHD. In the first study on myeloid cells performed during HIV-2 infection in Portugal, high levels of CD14^bright^CD16^+^ monocytes had been found during HIV-2 chronic infection compared to controls from the local blood bank in Lisbon, Portugal (20). These events corresponded to the intermediate monocytes and to part of the non-classical monocytes measured in our work. The female/male sex ratios were lower in our cohort than in the Portuguese cohort (1.83 or 1.25, respectively, vs. 2.22), but our global comparison of non-classical monocyte counts showing higher counts in females than in males did not contradict the conclusions of both studies. Thus, in the present study, HIV-2 did not have any impact on monocyte subpopulations when infected people were compared to geographically-matched controls. For conventional DC (cDC1 and cDC2) populations, measured for the first time in HIV-2-infected people, no statistical difference was found between GeoHD and HD, neither between PLWHIV-2 and GeoHD. In the Portuguese cohort, compared to local blood bank donors, low counts of myeloid DC, defined as (CD16, CD14, CD3, and CD20)-negative, CD123-negative, HLA-DR^+^, CD11c^+^ cells, had been found, and they correlated with viral loads and with plasma sCD14 levels. The ages of the participants were comparable (50 or 53 vs. 52 years), and our global cDC1% did not show any gender-related difference. Compared to the Portuguese cohort, the IMMUNOVIR-2 cohort had a similar proportion of participants with undetectable HIV-2 viremia (25/29 vs. 21/25), but with lower thresholds (40 or 100 vs. 200 copies/mL), lower maximal VL at sampling (117 vs. 26,263 copies/mL) and higher CD4 counts [LTNP: 895 CD4 T cells/μL, P: 502, number of participants with CD4 < 350/μL: 2, vs. in the Portuguese cohort 538 cells/μL and 1/3 of the participants with CD4 < 350/μL (20)]. Thus, the MMUNOVIR-2 cohort had a lower global progression than the Portuguese cohort, which may explain the absence of defect in DC population numbers, even though they were defined more precisely.

As we had found high plasma sCD14 only in HIV-1 infected individuals with high viremia (13), and as HIV-2-infected participants from the IMMUNOVIR-2 study had undetectable to low viremia, we were not surprised that they had normal translocation markers compared to GeoHD. Neither DC or monocytic population counts nor plasmatic markers of bacterial translocation were correlated in this study to viral loads, proviral loads or to progressor status. This is comparable to the results previously obtained in the larger ANRS CO5 cohort, where no differences in sCD14 plasma levels were found between progressors and non-progressors, although sCD14 levels were predictive of progression (36). Differently from the HIV-2 infected Portuguese cohort (20), we did not find any correlations between plasma bacterial translocation markers and DC counts. However, we found correlations between inflammatory monocytes expressing surface CD16 (intermediate, non-classical or slan^+^ monocytes) and plasmatic markers of bacterial translocation, i.e., LBP or sCD14 levels, like in former studies in PLWHIV-1 (11). We showed previously that CD14^bright^ classical monocytes can differentiate *in vitro* into slan^+^ monocytes in the presence of M-CSF and GM-CSF (13), the latter occasionally found to be detectable in the plasma during acute or chronic HIV-1 infection (40, 41). However, in the plasma from all of the HIV-2 infected participants studied here, GM-CSF levels were undetectable. Similarly, this cytokine remained undetectable in the plasma of all but one GeoHD. The distinction between P and LTNP is rarely made in HIV-2-infected cohorts despite its potential interest. The correlations between inflammatory monocyte counts and plasma LBP levels as a sign of microbial translocation found in all HIV-2 infected donors (LTNP + P) were sustained in LTNP alone, and not in P alone. This is counter-intuitive, as microbial translocation and plasmatic inflammation markers are known to increase with age and with HIV-1 infection independently (28). This may be due to the low number of progressor participants, as some of them were in fact non-LTNP, with a relatively low level of HIV VL compared to other cohorts, as discussed above. Alternatively, a protective role might be attributed to inflammatory monocytes (defining here CD16^+^ monocytes) against the progression of HIV-2 infection toward AIDS, i.e., in terms of viral loads or CD4 T cell counts. Indeed, these cells have antibody-dependent cellular phagocytic and cytotoxic effector functions (42). Therefore it is possible that despite apparent integrity of the intestinal mucosa (34), microbial translocation may occur more often in HIV-2 infected individuals than in healthy donors, inducing stimulation of classical monocytes by cytokines locally (without systemic dissemination of GM-CSF), sCD14 shedding, and CD16 and slan surface expression. Thus, circulating inflammatory monocyte counts may be a sensitive sign for intestinal mucosa intermittent leakage.

One very interesting lesson from this study is the comparison of the two control groups. The counts of circulating inflammatory non-classical monocytes, and especially slan^+^ monocytes, were significantly higher in the GeoHD group originating from West-Africa than in the HD group. The HD control group matched for sex and age with HIV-2 infected individuals was recruited at a local blood bank, as usual in many HIV-2 infection studies. French legal rules require that blood banks do not collect any data related to ethnicity. However, the origins of the population in the Paris area are very diverse. Here, for the IMMUNOVIR-2 participants and after specific approval from the ethical board, we collected data on birth country, nationality (original or acquired) and the country where the contamination occurred. A similar effort was done in the Portuguese cohort by the classification into “black” or “white” individuals, which did not give rise to any difference between the two groups for plasma LPS or for any of the data levels (20). In other studies, higher percentages of CD14^+^CD16^+^ monocytes and lower percentages of CD14^+^CD16^−^ monocytes were found in Africans than in Caucasians, perhaps in relation with former exposure to pathogens such as *Plasmodium falciparum* and *Schistosoma haematobium* (43). Higher frequencies of CD14^+^CD16^+^ “proinflammatory” monocytes, with higher expression of HLA-DR and PD-L1, were also found in a cohort of 50 healthy volunteer from Entebbe, Uganda than in a cohort of 50 healthy volunteers of similar ages from Lausanne, Switzerland (44). This difference was not related to gender differences. It was accompanied by higher CD16^+^HLA-DR^+^ “exhausted” NK cell frequencies, higher effector memory CD4^+^ and CD8^+^ T cell frequencies, and a more activated B cell compartment. Importantly, proinflammatory monocyte frequencies correlated negatively with these volunteer's neutralizing antibody responses to a live virus vaccine, the licensed yellow fever vaccine 17D (YF-17D). Other reports on adaptive or innate immunity during HIV-2 infection were performed in West Africa, with local controls (4, 45, 46). Comparisons of innate immunity parameters during HIV-1 infection were also performed between different countries. For instance, significantly higher plasma sCD14 levels were found in cART-naive HIV-1-infected donors from Mexico compared to similar donors from South Africa, along with other differences in inflammatory biomarkers (47). Untreated, PLWHIV-1 from Entebbe, Uganda, were found to have low CD11c^+^ and CD11c^−^ DC counts like PLWHIV-1 from the UK, compared to non-African laboratory workers from the UK (48). These cohorts recruited before the cART era had high viral loads and low circulating CD4 T cell counts. It was claimed that in Africa, baseline sCD14 plasma levels were lower, and LBP similar, in 86 HIV-uninfected donors from Uganda (who seroconverted and were studied longitudinally) compared to HIV-uninfected donors from the US (median 53 years old, 62.5% African-Americans, 50% female), and that they did not change significantly during HIV-1 infection (49). This was contested by other studies finding microbial translocation during HIV-1 infection in Kenya (50) or in India (51).

Origin differences, whether between countries but also between socio-economical groups, may have an impact on innate immunity baseline and pathological parameters through many factors. (1) Gender induces differences depending either on gene products or on hormones (39). (2) Aging clearly has an impact on microbial translocation and inflammation (28). (3) Coinfections with opportunistic or other pathogens, hygiene, or microbial and climatic environment and (4) Medications (antibiotics, anticancer drugs, immune suppressors, and proton pump inhibitors.) may also induce microbial translocation. (5) Food and substance intake, particularly alcohol intake (52) and high fat diet, are known to have a direct impact on microbial translocation and on the emergence of inflammation and metabolic syndrome, as shown experimentally in non-human primate SIV infection models (53). (6) All these factors may also affect the development of the immune system during early life, including fetal life, with differential expansion and polarization of adaptive and innate immune cell populations, compartment seeding and epigenetic imprinting. (7) Even epigenetic imprinting from previous generations may influence the innate immune response.

Our study has several strengths including (1) the choice of a cohort of donors from the same geographical origin as most PLWHIV-2, i.e., West Africa, and its comparison to a state-of-the-art control cohort from the local Blood Bank, as usually done in non-West African studies; (2) the identification of long-term non progressors compared to progressors in the HIV-2 infected cohort; (3) the homogeneity of the HIV-2 cohort, all asymptomatic, tested before any antiretroviral treatment, and all without opportunistic infections like tuberculosis, which could have confounded inflammatory parameters (47); (4) the precise delineation of monocyte and cDC subpopulations yet unstudied during HIV-2 infection, (5) the correlation of their frequencies with plasmatic microbial translocation markers. Inversely, the limitations are the low numbers of the donors, particularly from the same sex, due to the changing recommendations of treatment during the inclusion period (2013–2015, protracted until the end of 2018), and the legal impossibility to know the origin of the healthy donors from the Parisian Blood Bank.

In conclusion, this study underlines that the choice of specific controls, although never perfect, helps refine studies in specific populations. It shows higher counts of inflammatory monocytes, and especially slan^+^ monocytes, in healthy donors of West-African origin living in the Paris area, than in healthy donors from the local blood bank. In HIV-2-infected long term non progressor participants, and not in controls, these inflammatory monocyte counts correlated with plasmatic markers of microbial translocation, indicating a potential innate immune response to subclinical bacterial translocation, and therefore a potentially sensitive and early marker for inflammation linked to HIV-2 infection.

## Materials and Methods

### Participant Blood Samples

Blood samples (Table 1) were collected from participants who reside in the Paris area. Twenty-nine participants were part of the ANRS IMMUNOVIR-2 study (part of the French ANRS HIV-2 CO5 cohort). These participants were adult, asyptomatic, treatment-naive individuals infected with HIV-2 alone. Nineteen were non-progressors (LTNP; 11 females, eight males, median age 50 years, range 21–66 years), with asymptomatic HIV-2 infection ≥8 years, with at least three CD4 cell counts or plasma viral load (pVL) measures during the 5 past years, stable CD4 cell counts (CD4) ≥ 500/mm^3^ since at least 5 years without a rapid decrase in the CD4 cell count slope (i.e., >50 cells/year) during the last 3 years). Among LTNP, 17 originated from West-Africa, i.e., either Ivory Coast, Republic of Guinea, Guinea Bissau, the Gambia, Ghana or Senegal. Ten were non-LTNP (P) (five females, five males, median age 54 years, range 41–70 years), also treatment-naive, except for mother-to-child HIV transmission preventive treatment. Among P, 9 originated from West-Africa. Exclusion criteria were: Ongoing opportunistic infection or malignant disease. Moreover, blood samples were collected from 30 age- and sex-matched healthy controls, seronegative for HIV-1 and HIV-2. Ten of these controls (GeoHD; six females, four males, median age 42 years, range 29–48 years) originated from a similar geographic area as most PLWHIV-2, i.e., West-Africa, based on the collection of the following data: birth country, nationality, presumed country where contamination occurred. Sixteen other controls were also recruited by the blood bank (EFS-Saint-Antoine-Crozatier) and analyzed as controls, within an ethical convention with Inserm (seven females, nine males, median age 46 years). Exclusion criteria for all groups were: Age <18 years, Hb < 10g/dL in the past blood check less than a month before inclusion, known evolutive neoplasia, juridic incapacity. The study was approved by the ethics committee Comité de Protection des Personnes Ile de France XI. All subjects gave written informed consent in accordance with the Declaration of Helsinki. Plasma were collected and kept frozen. Blood counts were performed by Coulter counting. Peripheral blood mononuclear cells were isolated by Ficoll density gradient and labeled immediately.

### Flow Cytometry

Peripheral blood mononuclear cells were washed with cold PBS, stained with LiveDead (Life Technologies, 30 mn on ice), before adding 5% human serum (Sigma, 15 min), washed with Staining buffer (PBS, EDTA 2 mM, BSA 0.5%) and labeled in Staining buffer (20 min on ice), then washed, fixed in PBS 4% Paraformaldehyde. The following monoclonal antibodies were used: CD3-QDot605 (clone UCHT1 1/150) from Invitrogen; M-DC8-FITC (DD-1, 1/20), CD141(BDCA-3)-APC (AD5-14H12, 1/150) and CD303(BDCA-2)-PE (AC144, 1/10) from Miltenyi Biotec; CD1c(BDCA-1)-Pacific Blue (L161, 1/400; Biolegend); CD14-QDot655 (TüK4, 1/100; Invitrogen), CD19-ECD (J3-119, 1/10; Beckman Coulter), CD11c-AlexaFluor700 (3.9, 1/10; eBioscience); HLA-DR-PerCP (G46-6, 1/10), CD16-APC-H7 (3G8, 1/40), CD56-PE-Cy7 (NCAM 16.2 1/100), and CD45-Amcyan (2D1, 1/25) from BD Biosciences. Cells were washed with permeabilization buffer before flow cytometry and analysis (LSR II, BD; FlowJo v10.1, TreeStar, USA). Data acquisition and analysis were performed at the Cochin Cytometry and Immunobiology Facility. The absolute numbers of cells per blood microliter were calculated as before (13, 14, 21, 54, 55) by multiplying the Coulter blood count (performed independently on whole blood collected during the same blood sampling) of mononuclear cells (monocytes + lymphocytes), expressed as cells/μL, to the ratio [events for the population of interest/(lymphocyte + monocyte) events], expressed as a percentage, from flow cytometric event counts (Supplementary Figure 1).

### Multiplex ELISA

sCD14, sCD163 LBP, and GM-CSF concentrations were measured in duplicates in the plasma using Human magnetic Luminex assays (Biotechne, R&D, Lille, France) at the Cochin Cytometry and Immunobiology Facility.

### Statistics

The data from the different groups of donors were analyzed using the Kruskall-Wallis test with Dunn's post-tests correction for multiple testing, and the Mann-Whitney test when there were only two groups to compare. Chi square test was used to analyse proportions of female vs. male participants. Correlations were analyzed by Spearman tests. Differences were defined as statistically significant when *p* < 0.05. The Graphpad Prism v6.10 for Mac OS X was used.

## Data Availability Statement

The raw data supporting the conclusions of this article will be made available by the authors, without undue reservation.

## Ethics Statement

The studies involving human participants were reviewed and approved by Comité de Protection des Personnes Ile de France XI. The patients/participants provided their written informed consent to participate in this study.

## Author Contributions

AH, MI, JM, RC, and the IMMUNOVIR-2 group contributed to the conception and design of the study. NC and SM organized the database. MI, SI, MN, and AH performed the FlowJo and statistical analysis and drew the figures. MI and AH wrote the manuscript. MI, SI, JM, J-BG, KB, MA, SA, LVa, EH, SF, LVi, AH, and BC performed the labeling and FACS acquisition. KB performed the Luminex assays. SM is the PI of the ANRS-CO5 cohort. All authors contributed to IMMUNOVIR-2 brainstorming, manuscript revision, and they read and approved the submitted version.

## Conflict of Interest

The authors declare that the research was conducted in the absence of any commercial or financial relationships that could be construed as a potential conflict of interest.

## Supplementary Material

Supplementary Figure 1 FACS gating strategy for DC and monocyte enumeration.

Supplementary Figure 2 Correlations between monocyte counts and proviral loads.

Supplementary Figure 3 Effect of sex on cell numeration.

## Abbreviations

cDC: conventional dendritic cells (type 1, cDC1; type 2, cDC2)
EFS: Etablissement Français du Sang (French Blood Bank)
GeoHD: healthy donors matched for geographical origin with PLWHIV-2 from the LTNP and P groups
HD: healthy donors (the abbreviation in the text is for healthy donors from the blood bank (EFS)
LBP: Lipopolysaccharide-binding protein
LTNP: long-term non-progressors
P: progressors
PLWHIV: people living with HIV
WA: West-African.
