# Supplementary material for: Conventional Dendritic Cells and Slan+ Monocytes During HIV-2 Infection
Source: Front Immunol. 2020 Aug 13;11:1658. doi: 10.3389/fimmu.2020.01658 (PMC7438582; doi:10.3389/fimmu.2020.01658)
Supplement: Supplementary Figure 1 — FACS gating strategy for DC and monocyte enumeration. [file Data_Sheet_1.pdf]

Sup Fig 1 FACS gating strategy used for flow cytometric analysis

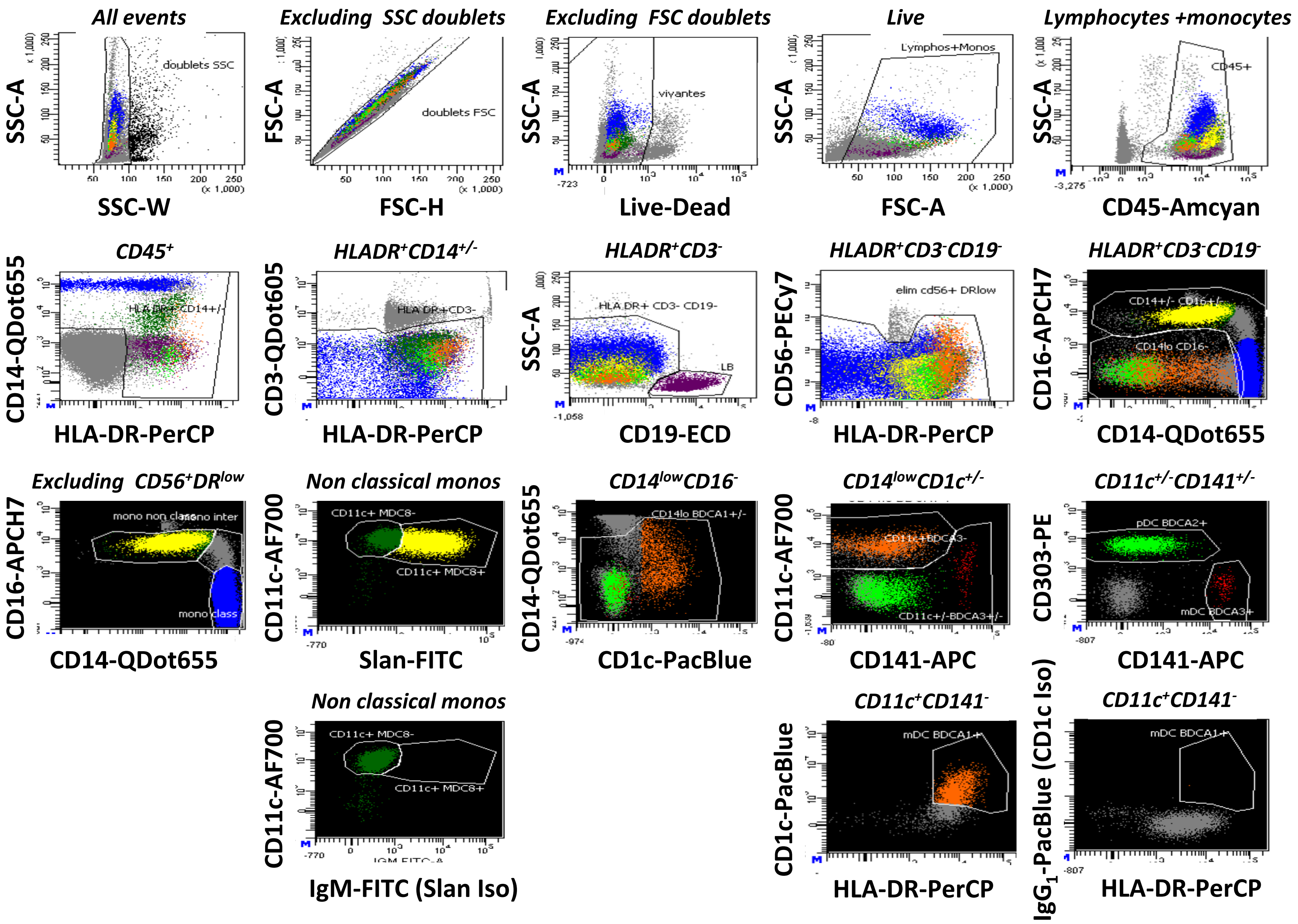

Sup Figure 2.

Correlations between monocyte counts and proviral loads

Spearman correlation r and p are indicated in blue

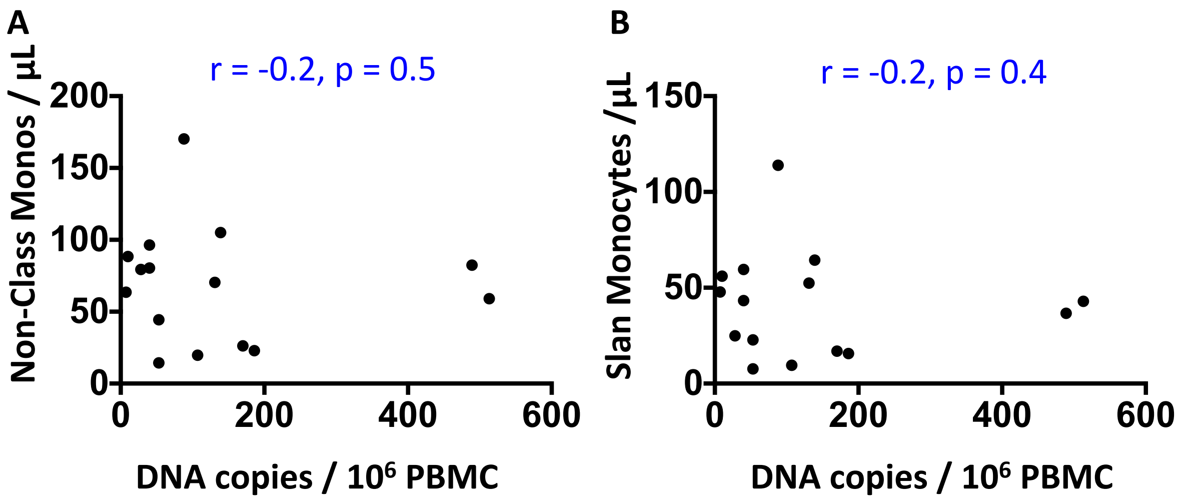

Sup Figure 3. Effect of Sex on Cell numeration

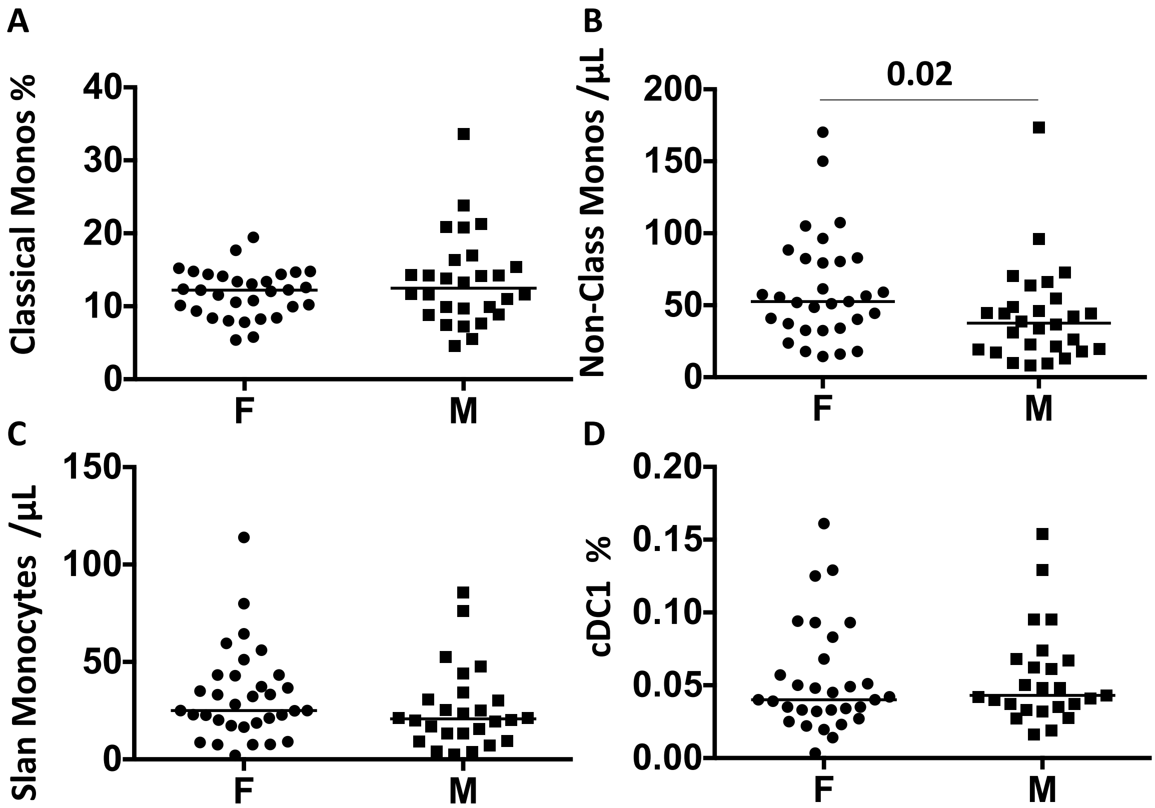

The % of classical monocytes and of cDC1 and the counts/µL of non-classical monocytes and slan-monocytes in all the HIV-2 infected or uninfected individuals studied here were compared between female (F) and male (M) donors using the Mann-Whitney test.
